# Clinically significant genomic alterations in the Chinese and Western patients with intrahepatic cholangiocarcinoma

**DOI:** 10.1186/s12885-021-07792-x

**Published:** 2021-02-12

**Authors:** Shifeng Xu, Yuan Guo, Yanwu Zeng, Zhijian Song, Xiaodan Zhu, Ning Fan, Zhilei Zhang, Guibing Ren, Yunjin Zang, Wei Rao

**Affiliations:** 1 Department of Hepatobiliary Surgery, Shandong Provincial Hospital affiliated to Shandong First Medical University, Shandong, China; 2 Liver Disease Center, The Affiliated Hospital of Qingdao University, Qingdao, China; 3 Origimed, Shanghai, China; 4 Organ Transplant Center, The Affiliated Hospital of Qingdao University, Qingdao, China; 5 Liver Disease Center, The Affiliated Hospital of Qingdao University, Qingdao, China; 6 Department of Hepatobiliary Surgery, The Fourth Hospital of Hebei Medical University, Hebei, China; 7 Oncology Department, The Armed Police Characteristic Medical Center, Hebei, China; 8 Organ Transplant Center, The Affiliated Hospital of Qingdao University, Haier Road No. 59, Qingdao, 266000 Laoshan District China

**Keywords:** Intrahepatic cholangiocarcinoma, Genomic alteration, Population, Clinical significance, Driver gene, Actionability

## Abstract

**Background:**

The goal of this study is to disclose the clinically significant genomic alterations in the Chinese and Western patients with intrahepatic cholangiocarcinoma.

**Methods:**

A total of 86 Chinese patients were enrolled in this study. A panel of 579 pan-cancer genes was sequenced for the qualified samples from these patients. Driver genes, actionability, and tumor mutational burden were inferred and compared to a cohort of Western patients.

**Results:**

Totally**,** 36 and 12 driver genes were identified in the Chinese and Western cohorts, respectively. Of them, seven driver genes (*IDH1*, *KRAS*, *TP53*, *BAP1*, *PBRM1*, *ARID1A,* and *NRAS*) were shared by the two cohorts. Four driver genes (*SPTA1*, *ARID2*, *TP53*, and *GATA1*) were found significantly correlated with the tumor mutational burden. For both cohorts, half of the patients had actionable mutations. The two cohorts shared the most actionable genes but differed much in their frequency. Though *KRAS* mutations were at the first and second actionable rank respectively for the Chinese and Western populations, they were still at a relatively low level of actionable evidence.

**Conclusions:**

The study on the clinical significance of genomic alterations directs the future development of precision medicine for intrahepatic cholangiocarcinoma treatment.

**Supplementary Information:**

The online version contains supplementary material available at 10.1186/s12885-021-07792-x.

## Background

The Chinese population has a higher incidence rate of intrahepatic cholangiocarcinoma (iCCA) in comparison to the Western population [1]. However, the incidence rate in the Western population is now rapidly increasing. Patients with iCCA regularly have poor prognosis. In a cohort study [2], half of the patients who underwent curative resection had a 5-year survival rate at 19% and a median survival of 27.6 months. With the advent of precision medicine, targeted therapy and immunotherapy would essentially reshape the treatment of iCCA, especially for those unresectable patients.

To start targeted therapy or immunotherapy, genotyping is the first step. Zou et al. [3] reported a landscape of intrahepatic cholangiocarcinoma in the Chinese population by whole-exome sequencing. They revealed eight driver genes in 103 iCCA patients. Those genes were *TP53*, *KRAS*, *IDH1*, *PTEN*, *ARID1A*, *EPPK1*, *ECE2,* and *FYN*. Another study in the Western population conducted by Lowery et al. [4] compared intrahepatic and extrahepatic cholangiocarcinomas with a panel of 410 genes. By sorting the frequency of mutations in patients, they got eight commonly mutated genes in intrahepatic and extrahepatic cholangiocarcinoma including *IDH1*, *TP53*, *ARID1A*, *BAP1*, *KRAS*, *PBRM1*, *SMAD4* and *ATM*. Besides, there was also a 30-patients study comparing different types of biliary tract cancer with a 22-gene panel by Hogdall et al. [5]. They identified three significantly mutated genes including *ARID1A*, *TP53,* and *KRAS* in CCA (cholangiocarcinoma). All the above studies concentrated on the mutations in one population, neglecting the difference between populations. In considering the significant incidence difference between the Chinese and Western populations, it would be interesting to know the clinical significance of their shared and distinct genomic alterations.

In this study, we collected 86 samples from Chinese iCCA patients. Since different researches used different methods to call the driver genes, directly comparing the results from multiple pieces of research could be problematic. Thus, we used the same pipeline to analyze driver genes, actionability, and tumor mutation burden and compared them between the Chinese and Western cohorts.

## Methods

### Patient and sample preparation

This study was approved by the ethical committee of the Affiliated Hospital of Qingdao University and Shandong Provincial Hospital Affiliated to Shandong First Medical University. A total of 86 Chinese patients with intrahepatic cholangiocarcinoma were enrolled (ORI dataset). Each participant had provided written informed consent. Samples were collected from surgery after diagnosis.

### Sequencing

Sequencing was conducted in a CLIA/CAP-compliant Molecular Diagnostics Service laboratory of Origimed Co., Ltd. The detailed sequencing procedure was described as our previous work [6, 7]. Briefly, tissue samples were collected for each patient. KAPA Hyper Prep Kit (Illumina) was used to extract DNA. At least 50 ng of double-stranded DNA was required for further library construction. Barcodes were added to the segments for multiplex sequencing to reduce the false discovering rate. From both segments’ ends, 151 bp nucleotides were read on an Illumina Novaseq 6000 (Illumina, San Diego, CA). Two samples did not pass library quality control. Totally, 84 samples had been sequenced.

### Variation calling

A total of 579 pan-cancer genes (Yuansuo® from Origimed incorporation, Shanghai, China) were captured by targeted amplification. Variation calling methods were referred to that used by the MSK cohort (https://github.com/rhshah/IMPACT-Pipeline). Briefly, reads were mapped to the UCSC hg19 reference sequences using BWA MEM (version 0.7.9a) [8]. Redundant reads were de-duplicated. GATK [9] was used to recalibrate base quality. Variants were called by Mutect [10], Pindel [11] and Somatic Indel Detector [9]. Structural variants were identified by Delly [12].

### Driver gene identification

To remove the false positive mutations or passenger mutations, the driver genes were identified by MutSigCV [13] using the GenePattern web service [14]. The pre-build full exome coverage file, covariate file and genome file for hg19 from GenePattern web service were used. The *p*-values for all the panel genes were inferred by MutSigCV. Significantly mutated genes were determined by the *p*-values adjusted by the Benjamini & Hochberg method.

### Pathway analysis

The enriched KEGG pathway [15] and gene ontology were analyzed using the DAVID web service [16]. The significant items were determined by the false discovery rate with a threshold at 0.05.

### Clinical actionability annotation of the somatic mutations

Clinical actionable mutations were annotated with a python software MafAnnotator.py published by OncoKB [17]. The parameter, disease type, was set “CHOL”. Mutations were annotated with six levels, they were, “Level_1”, “Level_2A”, “Level_2B”, “Level_3A”, “Level_3B” and “Level_4” [17].

### Tumor mutational burden analysis

Tumor mutational burden (TMB) was calculated as the number of mutations multiplied by a adjust factor. The adjust factors were 1.1152 and 0.9738 for the two assays from the MSK dataset, and 0.7875 for the ORI dataset. The TMB difference between mutated and wild type patients was compared by the Mann-Whitney U test. The false discovery rate (FDR) was used to adjust the *p*-values.

### Public dataset

A dataset from a Western cohort was prepared by Zehir et al. [18] and downloaded from cBioportal [19]. This cohort enrolled 87 patients from multiple ethnicities with intrahepatic cholangiocarcinoma in the United States by Memorial Sloan Kettering Cancer Center.

### Statistical test

Fisher’s exact test was used for the frequency comparison between the two cohorts. The Benjamini-Hochberg procedure was used for the correction of multiple testing. Power estimate of the two patient cohorts was performed with an R package “exact2x2” [20].

## Results

### The landscape of somatic mutations among the Chinese and Western populations

A total of 86 Chinese patients with primary intrahepatic cholangiocarcinoma were enrolled in this study (ORI dataset). The characteristics of these patients were listed in Table 1. The median age at diagnosis of the Chinese patients was 59, ranging from 18 to 83 years old. For comparison, we also included a cohort dataset (MSK) curated by Zehir et al. [18], which consisted of 87 iCCA patients from the Western population. The median age at diagnosis of the Western patients was 65 years old, ranging from 37 to 79. The Chinese cohort had a younger age than the Western cohort at diagnosis (*p-*value = 0.024, Mann-Whitney U test).

**Table 1:** Clinical characteristics of patients with intrahepatic cholangiocarcinoma

Characteristics		ORI	MSK
Age	Median (range)	59 (18–83)	65 (37–79)
Sex	Male	48	35
	Female	38	52
Stage	I	29	0
	II	8	0
	III	22	0
	IV	3	58
	Unknown	24	29
Histological grade	Low	2	0
	Middle	37	0
	High	29	0
	Unknown	18	87

**Fig. 1:**
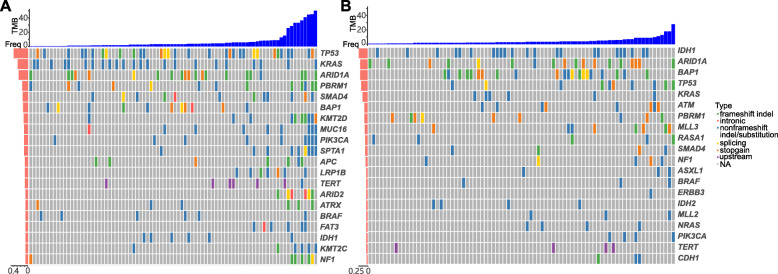
Mutational landscapes of iCCA patients from the Chinese and Western populations. **a** The mutational landscape of iCCA patients from the Chinese population (ORI cohort). **b** The mutational landscape of iCCA patients from the Western population (MSK cohort)

Samples from Chinese iCCA patients were sequenced by CLIA/CAP-compliant Molecular Diagnostics Service laboratory of Origimed Co., Ltd. Of the 86 samples, two samples did not pass the quality control and only the left 84 samples were sequenced. Figure 1 showed the top frequent mutations in the Chinese population and the Western population. The five top mutated genes in the Chinese population were *TP53*, *KRAS*, *ARID1A*, *PBRM1,* and *SMAD4*. The five top mutated genes in the Western population were *IDH1*, *ARID1A*, *BAP1*, *TP53,* and *KRAS*.

We also identified gene fusions in ORI with the same method as MSK cohorts. *FGFR2* fusions are most identified in both populations. There were 27 *FGFR2* fusion events in the ORI cohort and 33 in the MSK cohort. *FGFR2* was most fused with *WAC* in the ORI cohort and *BICC1* in the MSK cohort, respectively. Supplemental Table 2 lists all the fusion events in both populations.

### Driver genes in both populations

**Fig. 2:**
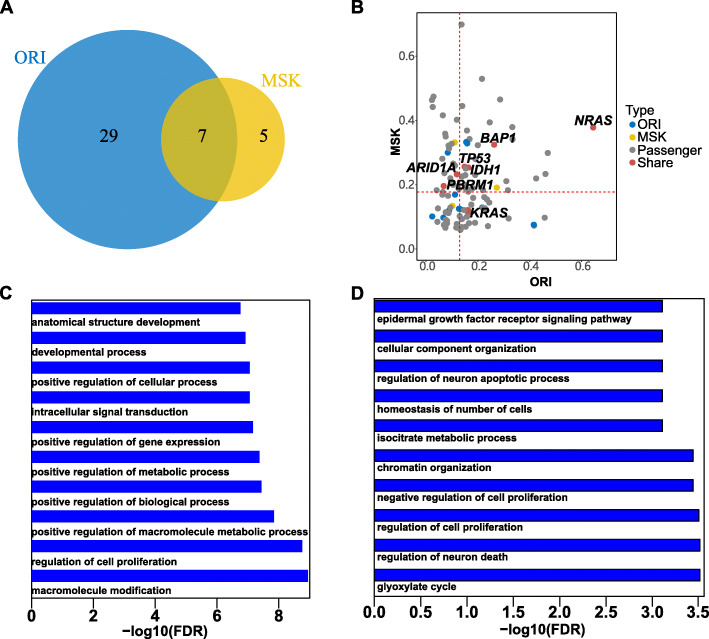
Significantly mutated genes in both populations. **a** The significantly mutated gene as revealed by MutSigCV analysis. **b** The median allele frequency (MAF) of mutations in ORI and MSK cohorts was plotted. The red dashes indicate the median value of MAF in ORI and MSK cohorts. **c** The biological process of gene ontology was enriched for the ORI cohort. **d** The biological process of gene ontology was enriched for the MSK cohort

To reduce the possible false positive, we utilized MutSigCV [13] to identify the driver gene. The same full genome coverage file was used as a control for both populations. There were 36 and 12 significantly mutated genes identified for ORI and MSK datasets, respectively (Fig. 2a) (Supplemental Table 1). Among them, seven mutated genes (*KRAS*, *TP53*, *BAP1*, *IDH1*, *PBRM1*, *ARID1A*, and *NRAS*) were shared. And, 29 and 5 driver genes were ORI-specific and MSK-specific, respectively. Most shared driver genes had a higher mutation allele frequency in both cohorts (Fig. 2b). Gene ontology analysis of the biological processes revealed enriched functions in macromolecule modification, regulation of cell proliferation, and positive regulation of metabolic process for the ORI cohort (Fig. 2c). MSK cohort was enriched in the glyoxylate cycle, regulation of neuron death and regulation of cell proliferation (Fig. 2d). KEGG pathway analysis identified significant pathways including melanoma, generic cancer and endometrial cancer in the ORI cohort (Supplemental Fig. 1) but none in the MSK cohort.

### Clinical actionability of genomic variations

**Fig. 3:**
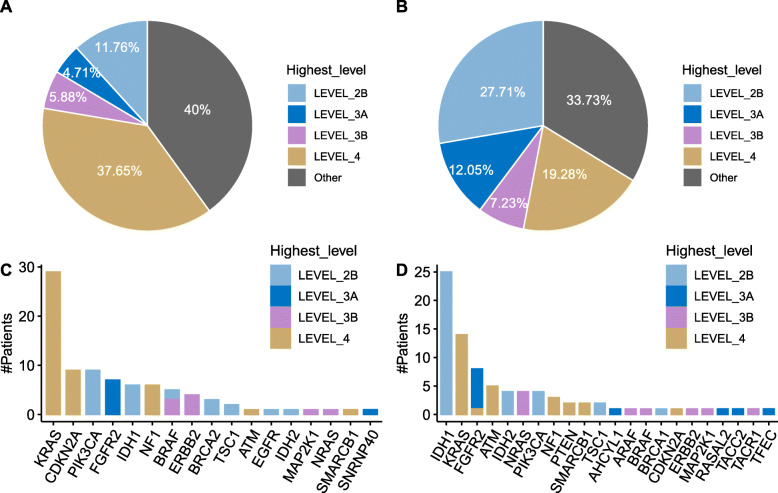
Clinical actionability of somatic alterations. **a** The percentage of patients was actionable in the ORI cohort. **b** The percentage of patients was actionable in the MSK cohort. **c** The number of patients was plotted against the best actionable genes in the ORI cohort. **d** The number of patients was plotted against the best actionable genes in the MSK cohort

To reveal the treatment potential, mutations were classified into six levels of drug actionability evidence proposed by OncoKB [17]. Each mutation was assigned a level according to the highest actionability proof strength. Then we summarized the best treatment for each patient. For the ORI cohort, 60% of patients had actionable mutations (Fig. 3a). For the MSK cohort, 66.27% of patients had actionable mutations (Fig. 3b). Among them, 11.76 and 27.71% of patients had standard care biomarkers predictive of response to an FDA-approved drug (Level_2B). Next, we studied whether the mutations in each gene had an equal actionability for both populations. The actionability for each mutation was summarized for each gene. The highest actionable level was chosen for each gene in a patient. Then we counted the number of patients for each gene by the highest levels. ORI cohort had top actionable mutations in *KRAS*, *CDKN2A*, *PIK3CA*, and *FGFR2* (Fig. 3c). MSK cohort had top actionable mutations in *IDH1*, *KRAS*, *FGFR2*, *ATM*, *IDH2,* and *NRAS* (Fig. 3d). Both cohorts had a similar set of actionable genes, differing only in their frequency (Fig. 3c and d).

### Mutation profile of typical genes

**Fig. 4:**
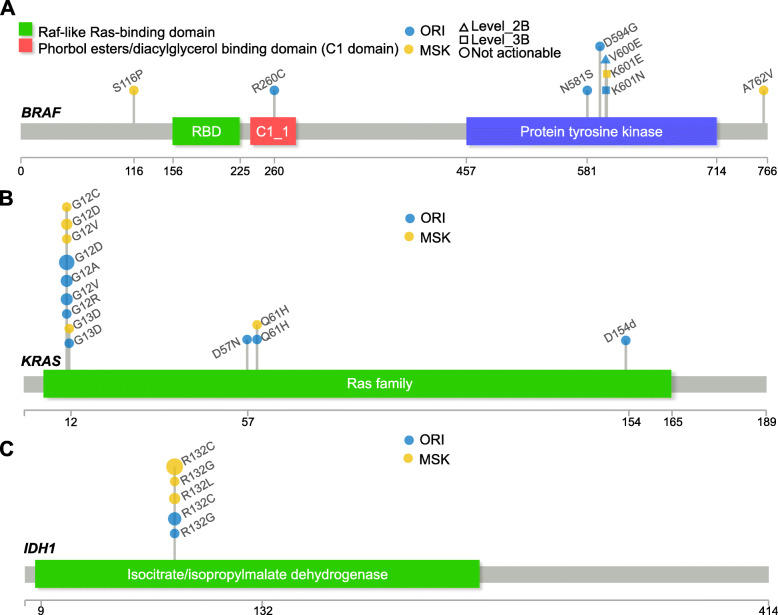
The mutation distribution of the actionable genes in ORI and MSK cohort. **a** The mutation distribution in BRAF. **b** The mutation distribution in KRAS. **c** The mutation distribution in IDH1

Mutation distribution on the important driver genes could result in different clinical outcomes and treatment efficacy. *BRAF* was such an example in the ORI cohort. As shown in Fig. 4a, *BRAF* had two mutations (V600E and K601N) in the patients of the ORI cohort with Level_2B and Level_3B actionability, respectively. *BRAF* in the MSK cohort only had one mutation (K601E) with Level_3B actionability. For ORI and MSK cohorts, the most actionable mutants were *KRAS* and *IDH1*, respectively. Their mutation distribution was depicted in Fig. 4b and Fig. 4c. *KRAS* had mutations at G12, G13, and Q61 in both cohorts. In the ORI cohort, *KRAS* also had the other mutations at D57 and D154. *IDH1* only had mutations at R132 with Level_2B actionability for both cohorts.

### Driver genes correlated with high tumor mutation burden

**Fig. 5:**
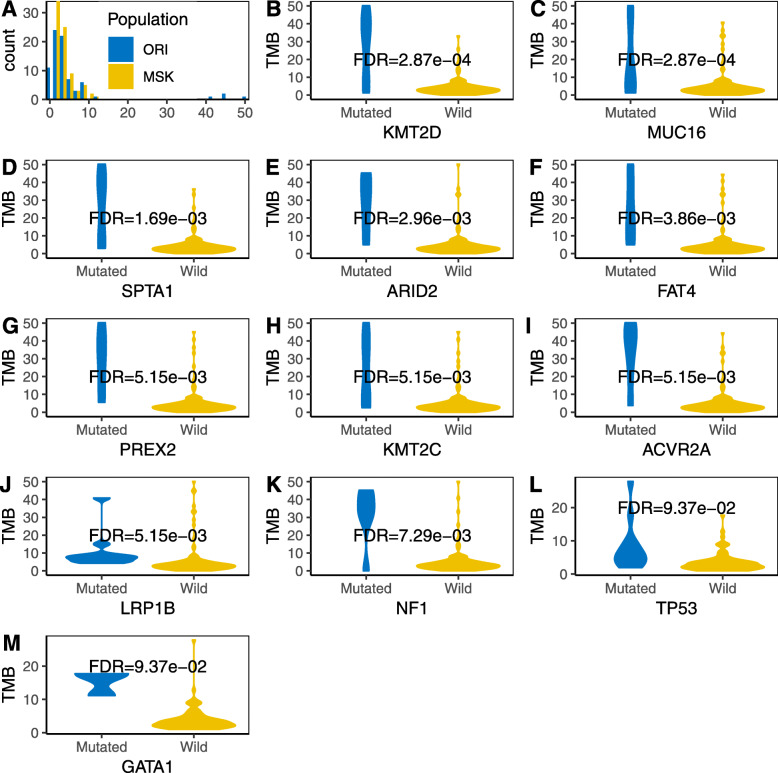
Genes associated with tumor mutation burden. **a** The tumor mutation burden (TMB) distribution in both cohorts was plotted. **b**-**k** Ten genes were significantly correlated with TMB in the ORI cohort (FDR < 0.01). **l**,**m** Ten genes were significantly correlated with TMB in the ORI cohort (FDR < 0.1)

TMB was considered a significant biomarker for immunotherapy efficacy in many other pieces of research. Next, we investigated the TMB distribution in both cohorts. First, the TMB was calculated based on the number of missense mutations across the covered genome length. The median TMB of the ORI cohort was 3.2, ranging from 0.6 to 51.4. The median TMB of the MSK cohort was 2.95, ranging from 0.98 to 27.88. No significant difference was found between the averages of the two cohorts (Fig. 5a). There were 11 and 5 patients with TMB > 10 mut/Mb for the ORI and MSK cohorts, respectively. Among them, two patients without actionable genes had TMB > 10 mut/Mb in each cohort. TMB was significantly correlated with *KMT2D*, *MUC16*, *SPTA1*, *ARID2*, *FAT4*, *FREX2*, *KMT2C*, *ACVR2A*, *LRP1B*, and *NF1* in the ORI cohort (Fig. 5b-k) and with *TP53* and *GATA1* in the MSK cohort (Fig. 5, l, m). Among those genes, *SPTA1*, *ARID2*, *TP53*, and *GATA1* were found as driver genes in their specific cohorts.

## Discussion

The significantly distinct incidence of iCCA between the Chinese and Western populations had driven us to disclose their genetic and actionable difference. Previously, driver genes were often identified by frequency among the populations. As suggested by Dees et al. [21] and Lawrence et al. [13], high-throughput sequencing studying could bring about many false-positive driver genes because of the mutational heterogeneity on the genome. To circumvent such errors, MutSigCV was employed. MutSigCV analysis had identified 36 significant mutated genes in the ORI cohort. The 10 most significant genes in the ORI dataset were *KRAS*, *TP53*, *SMAD4*, *BAP1*, *IDH1*, *PBRM1*, *ARID1A*, *SPTA1*, *NTRK3,* and *STK11*, among which *NTRK3* and *STK11* were not found in the top 20 highly mutated gene list.

Seven genes (*KRAS*, *TP53*, *IDH1*, *ARID1A*, *PBRM1*, *NRAS*, and *BAP1*) were shared between ORI and MSK cohort. Their mutation prevalence was higher than the median in both cohorts. We compared them to the results from other multiple publications [3, 13]. The sharing genes included *KRAS*, *TP53*, *IDH1*, and *ARID1A*, which were driver genes in both ORI and MSK cohorts. The other three genes (*PBRM1*, *NRAS*, and *BAP1*) were not reported in the research of Zou et al. [3] and Lawrence et al. [4]. Actually, *NRAS* is a very important gene involving many cancer-related signaling pathways, such as MAPK, mTOR, and PI3K-Akt signaling pathways. Currently, *NRAS* mutation is actionable at level 3B. Patients with *NRAS* mutation could be treated with Binimetinib or Binimetinib+Ribociclib.

Most iCCA patients can benefit from targeted therapy. About 60 ~ 70% of patients had actionable mutations in the ORI cohort and MSK cohort. For the actionable mutations, the MSK cohort had a higher actionable level than the ORI cohort, which implied a big potential in the treatment of iCCA for the Chinese population. Of these actionable mutations, *KRAS* and *IDH1* showed a significant bias in actionability between the Chinese and Western populations. The ORI cohort had a higher percentage of *KRAS* mutation at the actionable level 4 and less percentage of *IDH1* mutation at the actionable level 2B. Though *KRAS* mutations are not targetable directly, patients with *KRAS* mutation could benefit from inhibition of downstream effectors RAF and MEK. For example, selumetinib (MEK inhibitor) shows a good objective response for metastatic biliary cancer [22]. Considering the low evidence proof of *KRAS* mutations in both populations, drugs targeting downstream of *KRAS* should be improved urgently. Besides, we also identified fusion events and their actionability. Especially, *FGFR2* fusion events most happened in both populations. Targeting *FGFR2* fusion could bring about significantly better clinical outcomes. About 33.33 and 57.89% of patients with *FGFR2* fusions could be treated with target therapy at a high actionable level (Level 3A) in the ORI and MSK cohort, respectively. More *FGFR2* fusions should be further evaluated for the Chinese population.

Different mutations in the same gene could lead to different optimal treatments. ORI cohort had *BRAF* mutation V600E at a highly actionable level, 2B. V600E mutation in *BRAF* was recommended to be treated with Dabrafenib, Dabrafenib+Panitumumab+Trametinib, Dabrafenib+Trametinib, Encorafenib+ Binimetinib, Encorafenib+Cetuximab+Binimetinib, Trametinib, Vemurafenib, or Vemurafenib+Cobimetinib. MSK cohort had K601E mutation with a lower actionability (level 3B). K601E mutation in *BRAF* was recommended to be treated with PLX8394.

Immunotherapy also triggered much interest in iCCA treatment. In KEYNOTE-158 (NCT02628067; phase 2) and KEYNOTE-028 (NCT02054806; phase 1b) trials, immunotherapy has shown an improved PFS for advanced biliary cancer [23]. Even for refractory unresectable or metastatic biliary cancer, pembrolizumab also exhibited anti-tumor activity [24]. Several case studies also suggested its efficacy. To date, multiple immunotherapy trials are still under study [25]. The Food and Drug Administration even granted accelerated approval to pembrolizumab (KEYTRUDA, Merck & Co., Inc.) for any unresectable or metastatic tumors with high TMB (TMB > =10 mut/Mb) on June 16, 2020**.** Though the importance of immunotherapy has been widely accepted, how to improve clinical outcomes is still an urgent issue.

A Mann-Whitney U test revealed that TMB from both cohorts was not significantly different, which was consistent with the results of previous research [26]. It implied that the higher incidence of iCCA in the Chinese population should not come from a higher mutation rate and immunotherapy might have similar efficacy for both populations. Previously, there were several successful cases of immunotherapy for iCCA. Mou et al. [27] reported a late-stage iCCA Chinese patient with a high TMB. After a combination of anti-PD-1 immunotherapy and chemotherapy, the patient showed a remarkable response. Another Western patient with a high TMB, as reported by Gbolahan et al. [28], gained a good response by taking anti-PD-1 immunotherapy after chemotherapy resistance. Among ORI and MSK cohorts, there were two patients without any actionable mutation but with a higher TMB (> 10 mut/Mb) who could benefit from immunotherapy. Besides, the identified genes that were highly correlated with TMB could be biomarkers of immunotherapy prognosis. For example, MUC16 (CA-125) is a very long protein (14,500 amino acids), which is easier to mutate in cancer cells. *MUC16* was reported to associate with a higher TMB and a better immunotherapy outcome in gastric cancer [29].

This study aims to investigate the population-wide difference in the landscape of mutations and actionability. The 86 samples from the Chinese population were randomly collected, ignoring the potential subtype preference. The frequency of *KRAS* and *IDH1* mutation is similar to the other researches in the coordinate populations [3, 4]. The higher frequency of *KRAS* mutations in the Chinese population and *IDH1* mutations in the Western population could be associated with large duct and small duct iCCA [30, 31], respectively. It is noteworthy that no evidence supports discrimination of small duct from large duct iCCA in targeted therapy though it would be interesting to know whether or not duct types could work as a biomarker of clinical outcomes. We also found that *KRAS* and *IDH1* mutations showed no co−/exclusive occurrence among the ORI and MSK cohorts (Supplemental Fig. 2).

Mutational bias between the two cohorts could be caused by the difference in genetic background, endemic diseases, or special environmental factors. The Chinese population was more affected by liver fluke and hepatic-B virus while the Western population was by primary sclerosing cholangitis and chemical compounds [32–34]. Patients with IDH1 mutation were prone to be fluke-negative [35]. But in Western countries, it was still unknown about the risk factor for approximately 50% of patients [36]. Thus the risk factor of highly mutated IDH1 still needs further investigation. Similarly, KRAS mutations were only found associated with negative HBV without any confirmed risk factor [3]. Another highly mutated gene in the Chinese cohort, TP53, was found associated with liver fluke [35] and hepatic-B virus [3]. In summary, the known risk factors only accounted for a small part of the cases. More efforts are needed to resolve the puzzle.

This study also has its limitations. For example, TMB was calculated from different gene panels for the ORI and MSK cohort, which could weaken the results of the TMB comparison. But according to a more strict comparison, the conclusion still held between the Chinese and Western populations [26]. Besides, because iCCA has a low incidence and patients with it have a short survival time, we failed to enroll more patients to improve the statistical power. Supplemental Table 3 lists the power analysis result of the top 10 mutated genes in the comparison between the two cohorts. To avoid this limitation, we did not directly compare the proportion of each gene between the two cohorts but instead identify the driver genes with MutSigCV [13] and compared them. MutSigCV used patient-specific mutation frequencies and gene-specific background mutation rates, which can largely improve statistical power.

## Conclusions

In summary, this study compared the driver genes, actionability, and TMB in iCCA patients between the Chinese and Western populations. Shared and distinct driver genes were identified. *KRAS* and *IDH1* mutations account for about 20% ~ 30% of actionable targets with a significant bias in the two populations. The drugs targeting *KRAS* downstream pathway need urgent improvement. Half of the driver genes are still not actionable. The two populations did not show a significant TMB difference.

## Supplementary Information

**Additional file 1: Supplemental Table 1.** The actionability of driver genes

**Additional file 2: Supplemental Table 2.** Fusion events

**Additional file 3: Supplemental Table 3.** Comparison of mutational frequency for the top 10 mutated genes between the Chinese and Western cohorts.

**Additional file 4: Supplemental Figure 1.** The enriched KEGG pathways of ORI driver genes

**Additional file 5: Supplemental Figure 2.** The co−/exclusive occurrence mutations. (A) The co−/exclusive occurrence mutations in the ORI cohort. (B) The co−/exclusive occurrence mutations in the MSK cohort.

## Data Availability

The ORI dataset is not publicly available due to patients’ information protection. The MSK dataset is deposited on the cBioportal database (https://www.cbioportal.org/study/summary?id=msk_impact_2017).

## Abbreviations

iCCA: Intrahepatic cholangiocarcinoma
CCA: Cholangiocarcinoma
FDR: False discovery rate
TMB: Tumor mutational burden
