# Supplementary material for: Clinically significant genomic alterations in the Chinese and Western patients with intrahepatic cholangiocarcinoma
Source: BMC Cancer. 2021 Feb 12;21:152. doi: 10.1186/s12885-021-07792-x (PMC7879680; doi:10.1186/s12885-021-07792-x)
Supplement: Supplementary file 1 — Additional file 1: Supplemental Table 1. The actionability of driver genes [file 12885_2021_7792_MOESM1_ESM.docx]

**Supplemetal Table 1. The actionability of driver genes**

| Currently actionable driver genes in ORI | KRAS, IDH1, FGFR2, NRAS, BRAF |
| --- | --- |
| Currently nonactionable driver genes in ORI | TP53, SMAD4, BAP1, PBRM1, ARID1A, SPTA1, NTRK3, STK11, RNF43, MACC1, RB1, MLH1, RPTOR, PREX2, CFTR, TET3, DDR2, HRAS, SDHA, KEAP1, FGF4, PDCD1, FLT1, ATRX, ARID2, CAMTA1, SF3B1, SETBP1, FGF3, FGF23, KDM5B |
| Currently actionable driver genes in MSK | IDH1, KRAS, NRAS, IDH2, ATM |
| Currently nonactionable driver genes in MSK | TP53, BAP1, PBRM1, ARID1A, GATA1, RASA1, ABL1 |
