# Supplementary material for: Clinically significant genomic alterations in the Chinese and Western patients with intrahepatic cholangiocarcinoma
Source: BMC Cancer. 2021 Feb 12;21:152. doi: 10.1186/s12885-021-07792-x (PMC7879680; doi:10.1186/s12885-021-07792-x)
Supplement: Supplementary file 2 — Additional file 2: Supplemental Table 2. Fusion events [file 12885_2021_7792_MOESM2_ESM.docx]

**Supplemetal Table 2. Gene fusion events in ORI and MSK cohorts**

| **Fusion_1** | **Fusion_2** | **Cohort** |
| --- | --- | --- |
| FGFR2 | INA | ORI |
| MCL1 | intragenic | ORI |
| VEGFA | ZUFSP | ORI |
| C18orf8 | GATA6 | ORI |
| FGFR2 | NRAP | ORI |
| LOC102723493 | SMAD3 | ORI |
| KMT2D | PTPRK | ORI |
| intragenic | ERRFI1 | ORI |
| CTNNA1 | SIL1 | ORI |
| FGFR2 | intragenic | ORI |
| FGFR2 | WAC | ORI |
| KIF13B | ZNF703 | ORI |
| intragenic | KDM6A | ORI |
| intragenic | CDKN2A | ORI |
| intragenic | BRAF | ORI |
| RIN2 | LZTR1 | ORI |
| intragenic | SS18 | ORI |
| FOS | ZC2HC1C | ORI |
| RGS9 | PRKAR1A | ORI |
| intragenic | FAM135B | ORI |
| ZNF350 | PPP2R1A | ORI |
| FGFR2 | WAC | ORI |
| RASAL2 | ABL2 | ORI |
| FGFR2 | NRAP | ORI |
| FGFR2 | CFAP57 | ORI |
| SNRNP40 | FGFR2 | ORI |
| FGFR2 | CEP128 | ORI |
| FGFR2 | PHC1 | MSK |
| TFEC | FGFR2 | MSK |
| PIP5K1A | NOTCH2 | MSK |
| FGFR2 | BICC1 | MSK |
| PBRM1 | intragenic | MSK |
| FGFR2 | FAM13C | MSK |
| AHCYL1 | FGFR2 | MSK |
| RAD21 | intragenic | MSK |
| BRCA1 | intragenic | MSK |
| MAP3K1 | intragenic | MSK |
| TACR1 | ESR1 | MSK |
| PIK3C2G | RERGL | MSK |
| FGFR2 | BICC1 | MSK |
| FGFR2 | KIAA1217 | MSK |
| FGFR2 | BICC1 | MSK |
| STK11 | intragenic | MSK |
| RASAL2 | FGFR2 | MSK |
| TACC2 | FGFR2 | MSK |
| FGFR2 | NRAP | MSK |
