# Supplementary material for: Clinically significant genomic alterations in the Chinese and Western patients with intrahepatic cholangiocarcinoma
Source: BMC Cancer. 2021 Feb 12;21:152. doi: 10.1186/s12885-021-07792-x (PMC7879680; doi:10.1186/s12885-021-07792-x)
Supplement: Supplementary file 3 — Additional file 3: Supplemental Table 3. Comparison of mutational frequency for the top 10 mutated genes between the Chinese and Western cohorts. [file 12885_2021_7792_MOESM3_ESM.docx]

**Supplemental Table 3. Comparison of mutational frequency for the top 10 mutated genes between the Chinese and Western cohorts.**

| Gene | ORI.n | MSK.n | ORI.freq | MSK.freq | power | p.value | FDR |
| --- | --- | --- | --- | --- | --- | --- | --- |
| KMT2D | 11 | 0 | 0.127907 | 0 | 0.98919 | 0.000843 | 0.011805 |
| KRAS | 24 | 6 | 0.27907 | 0.068966 | 0.976158 | 0.002526 | 0.032841 |
| TP53 | 33 | 11 | 0.383721 | 0.126437 | 0.985701 | 0.003607 | 0.041013 |
| MUC16 | 9 | 0 | 0.104651 | 0 | 0.953842 | 0.003418 | 0.041013 |
| SPTA1 | 9 | 0 | 0.104651 | 0 | 0.953842 | 0.003418 | 0.041013 |
| MLL3 | 0 | 9 | 0 | 0.103448 | 0.953741 | 0.003556 | 0.041013 |
| IDH1 | 7 | 23 | 0.081395 | 0.264368 | 0.929918 | 0.009274 | 0.074195 |
| SMAD4 | 13 | 4 | 0.151163 | 0.045977 | 0.69775 | 0.042505 | 0.297533 |
| RASA1 | 0 | 5 | 0 | 0.057471 | 0.564715 | 0.059701 | 0.358209 |
| PIK3CA | 9 | 3 | 0.104651 | 0.034483 | 0.476893 | 0.134559 | 0.672797 |
| BAP1 | 11 | 20 | 0.127907 | 0.229885 | 0.476309 | 0.173292 | 0.693168 |
| ARID1A | 24 | 19 | 0.27907 | 0.218391 | 0.182576 | 0.499755 | 1 |
| PBRM1 | 13 | 11 | 0.151163 | 0.126437 | 0.081019 | 0.828093 | 1 |
| ATM | 5 | 9 | 0.05814 | 0.103448 | 0.197695 | 0.408072 | 1 |

Note: n, gene number; freq, frequency;FDR, false discovery rate.
