# Supplementary figures and images for: Clinically significant genomic alterations in the Chinese and Western patients with intrahepatic cholangiocarcinoma
Source: BMC Cancer. 2021 Feb 12;21:152. doi: 10.1186/s12885-021-07792-x (PMC7879680; doi:10.1186/s12885-021-07792-x)

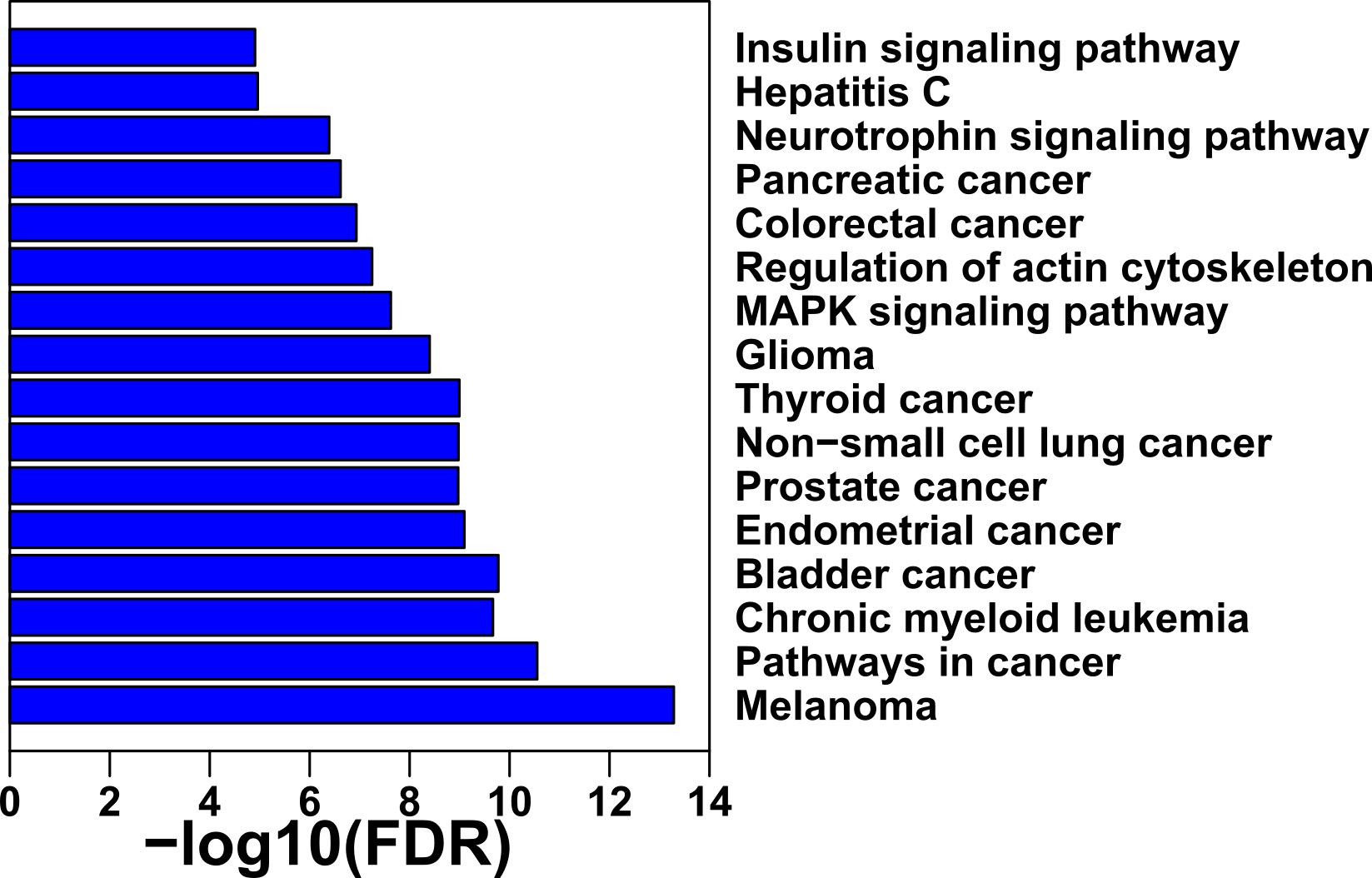

Supplement: Supplementary file 4 — Additional file 4: Supplemental Figure 1. The enriched KEGG pathways of ORI driver genes [file 12885_2021_7792_MOESM4_ESM.jpg]

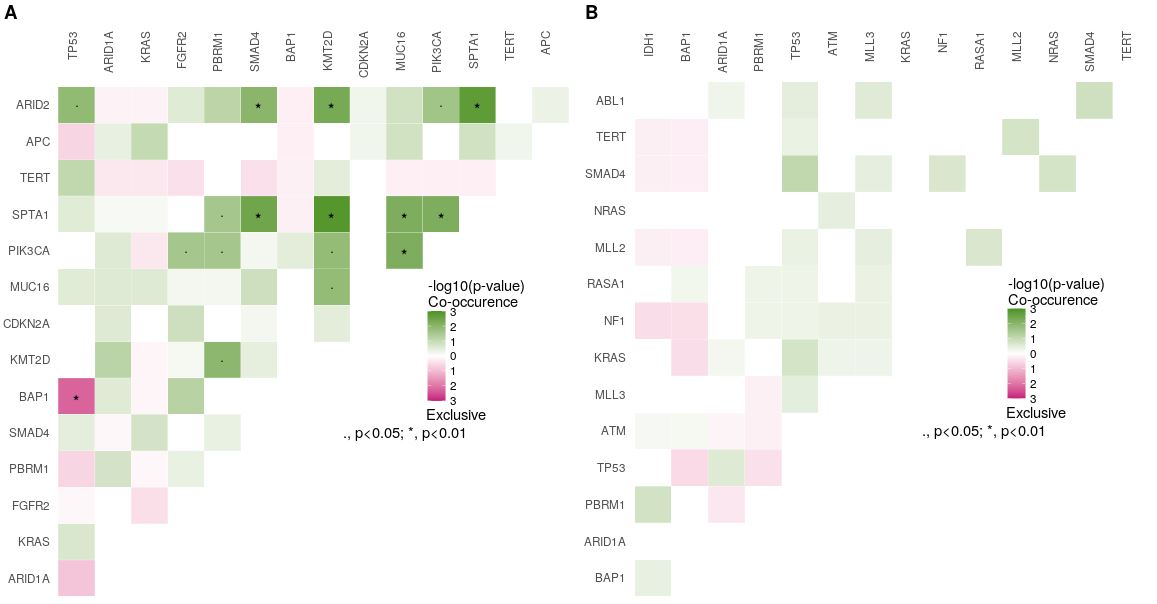

Supplement: Supplementary file 5 — Additional file 5: Supplemental Figure 2. The co−/exclusive occurrence mutations. (A) The co−/exclusive occurrence mutations in the ORI cohort. (B) The co−/exclusive occurrence mutations in the MSK cohort. [file 12885_2021_7792_MOESM5_ESM.jpg]
